# When the source is a bot: How people adapt their evaluation strategies to assess AI-generated content

**DOI:** 10.1371/journal.pone.0345300

**Published:** 2026-03-30

**Authors:** Shakked Dabran-Zivan, Inbal Klein-Avraham, Ayelet Baram-Tsabari

**Affiliations:** Faculty of Education in Science and Technology, Technion Israel Institute of Technology, Haifa, Israel; SUNY Downstate Health Sciences University, UNITED STATES OF AMERICA

## Abstract

Generative artificial intelligence (GenAI) blurs the boundaries between expert and non-expert sources, as it increasingly distributes and creates scientific content. This study examines how individuals adapt evaluation strategies, including content and source evaluation, and corroboration, when using GenAI versus a search engine. Based on performance tasks in which participants evaluated science-related socio-scientific dilemmas and follow-up interviews with 30 adult participants from diverse educational backgrounds, findings reveal that users employed these strategies on both platforms but adapted them in distinct ways. We identified two evaluation strategies that emerged as analytical constructs from the qualitative data. First, to corroborate output, participants frequently used a strategy we titled ‘representation evaluation,’ assessing whether GenAI accurately summarized its sources rather than verifying source agreement independently. Second, participants also applied ‘meta source evaluation,’ relying on their familiarity with sources provided by GenAI instead of directly evaluating the sources themselves. Although all participants engaged in dialogue with the chat, they did not leverage the bot’s dialogue capabilities to assess credibility, and many relied on a “machine heuristic”, assuming GenAI’s inherent correctness, reflecting a well-documented over-trust in automated systems. This research underscores the importance of developing and assessing critical evaluation skills for navigating AI-generated scientific information. Specifically, it extends existing models of online information evaluation to contexts mediated by artificial intelligence.

## 1. Introduction

The digital landscape through which we access, process, share, create, and communicate information is constantly developing, as are the skills required to navigate these environments effectively. Information evaluation skills are crucial, especially in today’s expanding digital landscape, where readers are often responsible for determining the trustworthiness of information with professional gatekeepers input becoming scarce.

Traditional models of credibility evaluation were developed for human-authored, source-transparent contexts, where cues such as identifiable authorship, institutional affiliation, or peer review help readers assess trustworthiness. However, the rapid integration of GenAI into everyday information practices challenges these models. When information is produced or mediated by AI systems, the origins of data, the reasoning processes, and the identity of the “source” are often opaque. This raises fundamental questions about how established evaluation strategies apply in AI-mediated environments.

According to dual process theory, people evaluate information using two systems: the intuitive and fast “System 1,” and the slower, more analytical “System 2” [1]. In online settings that favour speed and minimal effort, users often rely on System 1 thinking, making quick credibility judgments that are prone to cognitive biases such as confirmation bias, the tendency to favour information that supports existing beliefs while ignoring contradictions [2,12]. By contrast, System 2 thinking encourages deliberate, reflective analysis, which includes slowing down to question, verify, and compare information [3]. The literature identifies several evaluation strategies that require deeper cognitive engagement, including content evaluation, source evaluation, and corroboration with other credible sources [4,5].

Recent works emphasize source evaluation as the primary strategy for assessing complex scientific information. It acknowledges the epistemic dependence of non-experts, which refers to their reliance on trusted intermediaries because they cannot directly verify the accuracy of scientific claims [6]. The emergence of GenAI, however, presents a challenge to these strategies, since the source of the information is the GenAI itself, and its training data and content creation processes are often unknown to the users. Consequently, traditional cues of credibility are often absent. Not only that ‘flattening of expertise’ blurs the distinction between expert and non-expert voices, but it is also accompanied by an increasing user’ dependence on opaque systems whose reasoning they cannot inspect. These developments create new challenges in verifying or attributing knowledge and raise questions about how well-established evaluation strategies apply in AI-mediated information environments [7].

In the face of escalating misinformation, these challenges highlight the need to reconsider how people learn to evaluate AI-generated information. Beyond the conceptual understanding of its outputs, users and educators must also recognize GenAI’s epistemic limitations. These include the opacity of its algorithms, potential contamination or bias in its data sources [8], and being prone to ‘hallucinations,’ the generation of syntactically and contextually correct, reasoned, and confident answers that are nonetheless incorrect [9]. While research has investigated how people evaluate GenAI based on its fairness, responsibility, and transparency [10], studies examining the evaluation strategies used by people when engaging with GenAI are rare.

Toward this end, our aim is to examine how individuals adapt their existing evaluation strategies when engaging with GenAI compared to a popular search engine in everyday science-related dilemmas. We selected Microsoft’s Bing-Chat (today Copilot) as the GenAI platform, and Google Search was chosen as the comparison tool. This comparison is both theoretically and practically meaningful: Bing-Chat represents a generative-AI system that synthesizes and attributes information within a conversational interface, presenting its cited sources directly to the user, whereas Google Search functions as a retrieval platform that requires users to locate, navigate, and evaluate multiple external sources independently. In both environments, users have access to sources, yet the modes of interaction and presentation differ substantially. Contrasting these environments enables us to explore how users adapt their evaluation strategies. More specifically, we ask: how do users evaluate science-related information generated by Bing-Chat compared to that suggested by Google Search?

The findings of this study enrich the broader literature on information evaluation by revealing emergent evaluation behaviors that occur specifically in AI-mediated contexts. They expand existing frameworks of online information evaluation to account for the unique epistemic and interactional features of GenAI environments, offering valuable insights for both information science and science education.

The remainder of this paper is structured as follows: Section 2 reviews relevant literature on evaluating information in the online space and information evaluation in GenAI. Section 3 details the materials and methods. Section 4 presents the findings, and Section 5 discusses their theoretical and educational implications, followed by the study’s limitations and conclusion.

## 2. Literature review

### 2.1 Theoretical framework: A dual process of evaluating information in the online space

The dual system process theory suggests that when people evaluate information, they rely on a dual process, often referred to as ‘System 1,’ which is mainly intuitive, and ‘System 2,’ which is more analytical [1]. In fast-paced online environments, where minimizing time and cognitive load is the norm, users tend to develop rapid credibility evaluation strategies [11] – which theory would view as system 1. This reliance on quick judgments is particularly susceptible to the influence of cognitive biases [12], which affect people when evaluating information and making decisions. As a result of these biases, individuals may fail to fully gather, internalize, or systematically process information correctly, even when they have the expertise to do so [2]. Here we describe two cognitive biases, confirmation bias, which has been previously demonstrated in the context of online information evaluation, and the machine heuristic, which was demonstrated in the context of interactions with technology.

Confirmation bias is a relatively common phenomenon where individuals tend to search for, interpret, and remember information that confirms their beliefs while paying less attention to information that may contradict them. This bias can lead to a skewed search process, reducing exposure to information that conflicts with pre-existing beliefs [13]. The machine heuristic is a cognitive bias whereby people perceive automated machines as more objective and trustworthy than humans, although machines are vulnerable to errors and manipulation. This perception can lead to increased trust in machines, and studies have demonstrated how decision-making can be biased due to users’ over-reliance on automated machines [14].

In contrast, system 2 refers to analytical thinking and emphasizes the importance of slowing down the information evaluation process and delving deeper [3]. When people pause and thoughtfully consider the information their search revealed, they can better identify misinformation, even if it conflicts with their preferred outcomes and beliefs [15,16]. In the field of science education, growing emphasis has been placed on its role in equipping students with critical evaluation strategies to assess the reliability of scientific information, even on topics beyond students’ knowledge [17].

In the realm of the analytical “system 2” thinking, two approaches to evaluating scientific information are suggested: evaluating the content’s plausibility and assessing the source’s credibility [18]. Direct assessment of content accuracy reflects an awareness of the information itself. Plausibility is judged based on factors such as personal experience, subject knowledge, or logical consistency [5]. In contrast, assessing the source’s reliability and credibility employs strategies that do not require in-depth scientific understanding; they focus on determining whether the source can be trusted. This may involve scrutinizing the source’s reliability and/or integrity, or evaluating its expertise [5]. This method highlights our epistemic dependence on the expertise of others—the intellectual work, knowledge, and testimony provided by others [19,20]. Based on these ideas, Osborne and Pimentel [17] suggested the ‘fast and frugal’ linear technique to evaluate the trustworthiness of specific scientific claims based on judgments of credibility such as conflict of interest, relevant expertise and alignment with expert consensus.

One prominent technique for source evaluation is the lateral reading approach [21], which mirrors the techniques used by professional fact-checkers. Lateral readers ask fundamental questions such as: Who is behind this information? and What do other sources say? [21]. When encountering a new or unfamiliar website, lateral readers extend their inquiry by opening additional tabs and conducting parallel searches. This method enables them to validate the credibility of the original source by cross-referencing it with various trustworthy sources available online [22]. Pimentel [23] demonstrated that students who combine the fast and frugal technique with lateral reading, click restraint, and informed use of Wikipedia enhanced their ability to assess the credibility of online scientific sources and significantly differentiate between high- and low-quality information.

The ability to critically evaluate and integrate diverse sources to draw well-founded conclusions and develop meaningful insights is widely regarded as a key skill for the 21^st^ century [24]. Corroboration entails testing and evaluating information by cross-referencing it with information from trustworthy sources [4].

How are these evaluation strategies applied in everyday contexts across various technological platforms? Are they comparably effective? Is GenAI, which plays an active role in the creation and distribution of knowledge [25], changing the way people access scientific information online? Here, we examine how individuals employ and adapt evaluation strategies when engaging with GenAI compared to search engines to resolve everyday science-related dilemmas.

### 2.2 Information evaluation in GenAI

In the digital landscape of the 21st century, GenAI has emerged as a major source of online information, now integrated into the daily practices of millions of users [26]. These models can swiftly generate realistic text, images, and videos that closely mimic human-created content, thereby reshaping how information is produced and consumed [27]. This technological shift further complicates the information ecosystem [28]. Within this reconfigured environment, GenAI simultaneously supports and hinders informed use of information: it broadens its usefulness through personalization and automation, yet narrows it through opaque filtering mechanisms that users can rarely inspect or contest. Importantly, these affordances do not stem solely from the technology but from how users perceive, interact with, and ultimately legitimize the outputs of AI systems. As a result, AI enhances convenience and accessibility while also posing significant risks to informational diversity, transparency, and epistemic autonomy in everyday information practices [29]. Furthermore, it enables the widespread circulation of misinformation disguised as credible scientific knowledge [30,31].

These risks are amplified by the fact that GenAI systems prioritize fluency and speed over factual accuracy and lack the capacity to reliably distinguish between fact and fiction [31,32]. The resulting “AI hallucinations” have already produced tangible harms across domains, leading to customer confusion and financial loss [33]. Recent experimental evidence further shows that similar inaccuracies can emerge in political contexts, where AI-generated dialogues meaningfully shift voter preferences even when some of the information is inaccurate, highlighting a critical epistemic risk for democratic decision-making [34].

These risks of AI hallucinations and inaccuracies is further compounded by the reliance of AI systems on machine learning algorithms, often described as “black boxes”. The internal logic guiding how these systems learn and generate outputs is largely opaque to human understanding. This lack of transparency underscores the centrality of trust when interacting with AI-driven decision-making processes [35]. Trust in AI refers to a person’s willingness to accept vulnerability based on positive expectations of the system’s benevolence, integrity, and functionality [35]. Higher levels of trust have been shown to increase both adoption and acceptance of AI recommendations [35,36], even when those recommendations are incorrect [37].

These epistemic risks of AI-generated content are further compounded by how users interact with GenAI systems. Research indicates that while college students are aware of the potential limitations of information produced by GenAI, they often lack the motivation to verify its accuracy [38]. In addition, competency is also an issue. Post-secondary students in Hong Kong struggled to assess the accuracy and validity of information generated by these tools [39]. Machine heuristics may also influence credibility judgments, as users frequently perceive information produced by AI systems as more objective and reliable than that from other online sources [40].

In evaluating AI outputs, people rely on a range of indirect cues rather than systematic verification. Journalists, for instance, apply forensic verification techniques to AI-generated visuals, including reverse image searches, analysis of lighting and shadows, and metadata inspection [41]. Visual and technical cues also play a role in how lay audiences assess credibility. Users attend to graphic anomalies, behavioural inconsistencies, and overall content quality, and often corroborate these impressions with prior knowledge or external sources [42,43]. Notably, unlike other online environments, the minimalist, familiar chatbot interface of systems such as ChatGPT appears to exert little influence on users’ trust judgments, suggesting that conventional interface-based heuristics may be less effective in AI-mediated settings [44].

Taken together, these findings point to a fundamental shift in the epistemic conditions under which information evaluation now occurs. Within GenAI environments, information is generated through obscure algorithmic processes that often obscure, displace, or reconfigure conventional evaluation cues. It is an open question, however, how these changes interact with traditional evaluation practices, such as content or source evaluation and corroboration. Responding to these altered conditions, the present study examines how established evaluation strategies are maintained, adapted, or transformed when individuals engage with GenAI compared to a traditional search engine. The following section outlines the research design developed to investigate these adaptive processes.

## 3. Materials and methods

### 3.1 GenAI platform choice

This research focuses on GenAI, specifically large language models (LLMs), which can generate quasi-human responses to instructions and questions formulated in natural language. The study data were collected between June and August 2023. At that time, ChatGPT3.5 did not function optimally in [local language], did not provide references, and was limited in providing current information since its corpus was up to date only through 2021 [45]. Given our aim to compare the evaluation of information provided by GenAI with that of a search engine, we chose an AI that more directly lent itself to comparison, Microsoft’s Bing-Chat (now known as Copilot), released to the public in February 2023. At the time of data collection, it communicated well in the local language, had access to the internet enabling it to offer current and updated information and cited its sources [45]. Google Search (hereafter Google) was the chosen search engine, as it is the most widely used search engine globally, with a market share (and dominance) of approximately 82% [46] and 98% in [country] [47].

The use of Bing-Chat increases ecological validity by reflecting the available technological conditions at the time of data collection, but also poses a limitation to generalizability. GenAI systems differ in citation behavior, transparency, and interface design, which may influence users’ evaluation processes. Accordingly, findings should be interpreted in the context of Bing-Chat’s specific affordances, and future research should assess whether similar patterns emerge across other GenAI platforms.

### 3.2 Research population and research sample

When the data were collected, most of the country’s adult population was using the internet daily (81%) [48]. In 2023, among internet users, 77% had heard of but never used GenAI, and 16% had used it several times [7]. Of those who used GenAI, two-thirds used it to search for science-related information [49].

Against this general backdrop, we assembled a diverse sample using quota sampling [50], which included undergraduate students alongside people with high school-level education, both with and without science-related backgrounds and with and without algorithmic knowledge and experience. The quotas included the following groups: Bachelor’s degree students with academic experience in algorithmics (Computer science or data science students) both with (n = 5) and without (n = 5) science education academic level; Bachelor’s degree students with NO academic experience in algorithmics both with (n = 5) and without (n = 5) science education academic level (e.g., nutrition science students vs social work students); and High school graduates with NO academic experience in algorithmics or an academic level education but professionals with a scientific background and relevance (n = 5), e.g., medical secretary versus professionals with No scientific background (n = 5), e.g., dancer.

To target the specific groups we identified, a preliminary recruitment survey was distributed through universities, sports team forums, and acquaintances. Using the survey, we ensured that participants met the basic criteria established for the quotas. Of the initial 182 respondents, we drew a sample of 30 interviewees to fill the recruitment scheme of heterogeneity regarding education level (see S1 File).

The total sample size (N = 30) is consistent with established qualitative research norms for achieving thematic saturation. Recent methodological reviews indicate that meaning saturation in semi-structured interviews is typically reached after approximately 24 interviews, with theoretical saturation commonly achieved within 20–30 interviews across grounded and thematic designs [51]. The small quota groups (n = 5) were not used for comparative analyses but rather functioned as a sampling frame to ensure heterogeneity in participants’ educational, scientific, and algorithmic backgrounds. This structure allowed us to balance diversity in the sample with sufficient depth of data to support analytical adequacy across the dataset as a whole.

Data collection took place between 19/06/2023 and 20/08/2023. All participants provided informed consent: they signed a written consent form as part of the questionnaire, which was also read aloud to them during a Zoom session (participants shared their screen to display the form). Verbal consent was given and recorded during the Zoom session. Among the final sample, 63% were women, ages ranged from 18 to 39, with 27% in the 18–22 age range, 60% in the 23–29 age range, and 13% in the 30–39 age range. Sample characteristics are detailed in S2 File.

### 3.3 Data collection and research tools

**Fig 1:**
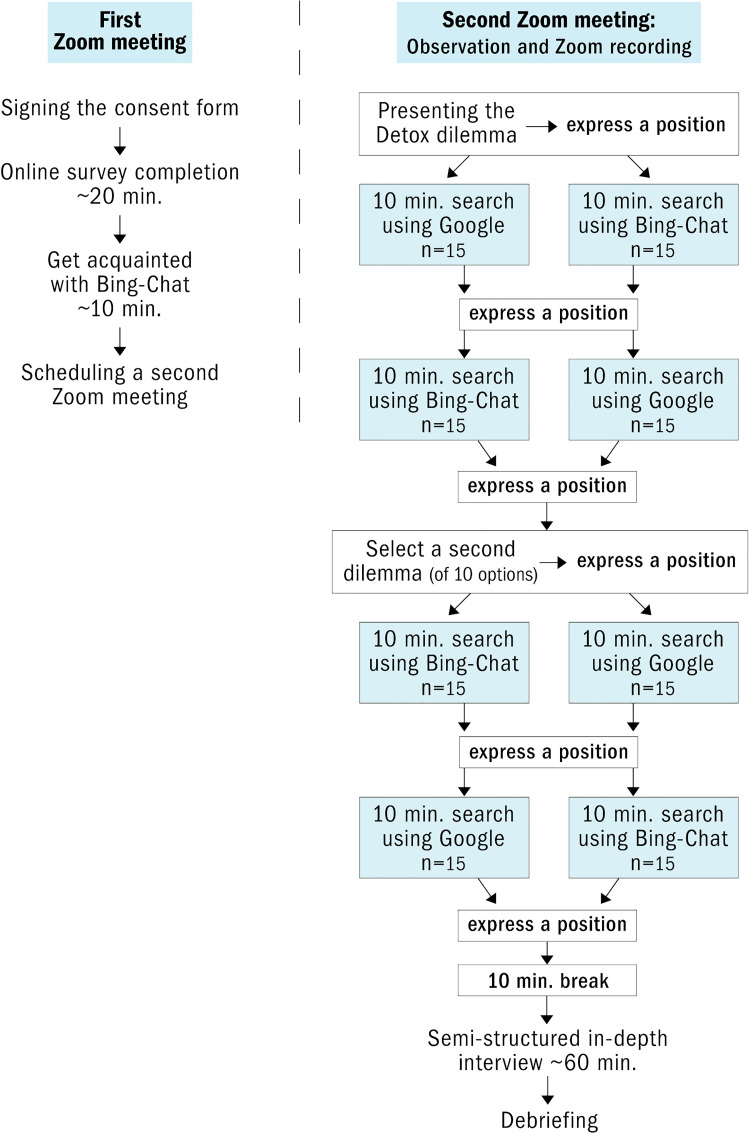
Research design setup.

This qualitative research combines a performance task, observation, and semi-structured interviews. The research setup consisted of two online meetings hosted on Zoom, recorded with the participant’s approval. For a full description of the research design setup, see Fig 1.

The first meeting introduced participants to Bing-Chat’s GenAI tools (see S3 File). The second session presented participants with two socio-scientific issues (SSI) since they are typically controversial and require evidence-based reasoning, providing a context for understanding scientific information [52].

The trade-off between participants’ personal interests and motivations and topic comparability poses a challenge for public engagement studies. To address this general problem, we employed two tasks in our study. The first task involved a shared topic across all participants to ensure comparability and control for topic-related variability. In contrast, the second task allowed participants to select a topic of their choice, thereby promoting authentic, interest-driven engagement. Each task offered distinct advantages, and together they provided a more comprehensive assessment of participants’ performance while using AI. This dual-task design strengthens the study’s internal validity by enabling the attribution of observed differences to the platform rather than to topic effects.

To ensure comparability and control for topic-related variability, in the first part of the meeting, all participants were introduced to the following vignette about Detox diets:

‘A close friend, whose health is important to her but who is experiencing financial difficulties, turns to you with a question: she is considering buying a detox kit based on a juice diet, which promises rapid improvement in physical health and mood. The downside is that it’s expensive. She wonders if the detox is safe and will be good for her. What would you recommend she do?’

To address anchoring effect, which is ‘relying too much on the first piece of provided information when making decisions’ [53], we asked participants for their opinion before asking them to search for information. Half of the participants then researched the topic using Bing-Chat and then on Google, while the others did their research in the reverse order. Participants were asked for their recommendation and basic justification three times: after reading the vignette, and after the first and second searches.

In the second part of the meeting, we asked participants to choose one of ten SSIs (vignettes are listed in S4 File, number of times each topic chosen in brackets, participants’ choices in S2 File): genetically modified food (n = 2), the use of disposable utensils (n = 9), children’s vaccines (n = 1), water fluoridation (n = 1), radiation from wireless internet routers (n = 5), wind turbines (n = 1), Ritalin for children (n = 7), cellular radiation (n = 1), hormone therapy during menopause (n = 1), and aluminium in deodorants (n = 2). Allowing participants to select their own topic supported authentic, interest-driven engagement. The SSI’s were chosen in a collaborative discussion of a bi-national team, thus holding relevance in at least two national contexts.

To address ordering effects, which occur ‘when the order of presented information alters user perceptions and decisions’ [53], those participants who searched first on Bing-Chat in the first part now searched first on Google (and vice versa). Participants were asked for their recommendation and basic justification three times: after reading the vignette, and after the first and the second searches.

While the participants performed the tasks, the interviewers (Authors 1 and 2) observed their process of searching for and evaluating the information (see S5 File). Finally, hour-long semi-structured interviews were conducted immediately after the performance tasks, focusing on the credibility and reliability of information sources and the use of evaluation strategies vis-à-vis the different technologies. Due to the unexplored nature of the research field, we aimed to minimize the influence of researchers’ preconceptions and expectations by employing a semi-structured interview format. This format balances a guiding framework with flexibility, enabling both broad understanding and in-depth insights [50] (see S6 File). When they finished, participants were presented with reliable information about the SSI they engaged with in a respectful debriefing to minimize risks.

### 3.4 Data analysis

Using ATLAS.ti, we conducted a reflexive thematic analysis following Braun and Clarke’s [54] conceptualization of thematic analysis as an interpretive, researcher-generated process. In this approach, themes are actively constructed through iterative engagement with the data, and analytic rigor derives from reflexivity, theoretical coherence, and depth rather than from coder agreement indices. Our analysis combined elements of directed and conventional qualitative content analysis [55]. Parts of the coding framework were inductively developed from meaning units within the dataset, while other sensitizing concepts were informed by prior research on online information evaluation. This dual orientation enabled close attention to both emergent practices and theoretically grounded constructs relevant to epistemic judgment in digital environments.

Each interview transcript and observation protocol was read multiple times to identify meaning units, phrases, or sentences that reflected evaluative actions or reasoning, which were then coded. Through iterative analytic meetings, the authors refined the coding scheme by merging, renaming, or discarding codes based on conceptual clarity, distinctiveness, and alignment with the study’s interpretive aims, consistent with Braun and Clarke’s [54] principle of design coherence. Once the coding scheme was agreed upon, the first author continued coding the full dataset, revisiting and adjusting codes as needed to maintain analytic coherence.

We examined thematic trends and commonalities across the dataset, drawing on observation data to capture enacted strategies during the performance tasks and interview data to illuminate participants’ reasoning. Triangulating these sources ensured that the resulting themes reflected coherent and well-grounded patterns. We compared emerging themes from observations and interviews with constructs identified in the literature to identify both established evaluation strategies and new strategies specific to GenAI-mediated information evaluation. Codes were iteratively refined to develop a comprehensive and distinct set of assessment strategies applicable across information technologies (for the final themes, see Table 1). This allowed us to examine how users evaluated science-related information generated by Bing Chat compared with information suggested by Google Search.

**Table 1:** The thematic coding of the study.

	Evaluation method	Explanation	Expression in search engine	Expression in GenAI
Critical evaluation best practices	Content evaluation	“What to believe?”: Assesses claims based on consistency or coherence with prior knowledge or by engaging in a critical analysis of its logical consistency [3]	Plausibility of the information	
			Alignment with prior knowledge	
			Agreement with expert knowledge	
	Corroboration	Corroboration with information that is available elsewhere [2]	Cross-referencing the information (observations)	Clicking the sources provided by Bing-Chat to verify the AI-generated content (observations)
			Pointing to the importance of cross-referencing information (interviews)	Pointing to the importance of cross-referencing the information provided by the chat with the sources it provides (interviews)
	Source evaluation	“Whom to trust?”: Assessing claims based on the reliability and credibility of the source [3]	Accessed official sources (observations)	Participants explain that they judge the credibility of AI as a source based on the appropriateness of the sources it references (interviews)
			Pointing to the importance of familiarity with sources (interviews)	
			Proactive search for information about the author (observations)	
			Pointing to the importance of the author’s expertise and experience (interviews)	
Cognitive biases	Confirmation bias	Individuals tend to search for, interpret, and remember information that confirms their beliefs while paying less attention to information that may contradict them	Searching for content that confirms my previous beliefs (observations)	
			The content confirms my previous beliefs (interviews)	
	Machine heuristic	People’s belief that automatic machines are more objective and more trustworthy than humans	–	Pointing that automatic machines are more objective and more trustworthy than humans (interviews)

Frequency counts are reported within the text when presenting the results and are also summarized visually.

### 3.5 Trustworthiness

Triangulation, by combining observations and interviews, helped to minimize bias and assess the comprehensiveness of participants’ responses [56] while increasing analytical validity. Peer debriefing was an integral part of the research process [57]. The research team held multiple consensus meetings at key stages of the analysis, once during coding and again during the synthesis of findings. When interpretive differences arose, we examined their sources in depth and resolved them through discussion until a complete consensus was achieved. The authors’ complementary expertise enriched these dialogues and contributed to a more nuanced co-construction of meaning, ensuring a coherent, transparent, and well-substantiated analytic process. In addition, throughout data collection, analysis, and interpretation, we consulted impartial colleagues who are experts in science communication and not co-authors of this paper. These experts reviewed the initial findings and preliminary analysis, providing critical feedback and guidance to help the researcher maintain integrity and foster a more reflective analysis [57]. Their input challenged the researcher’s assumptions and promoted methodological rigor [58].

### 3.6 Ethics

All participants were healthy adults who signed a consent form as a precondition for taking part in this study, and their identities were protected using pseudonyms. Participants were financially compensated for their time. The interview protocol was approved by the ethical institutional review board (IRB approval 2023−031).

## 4. Results

The evaluative patterns presented in this section are interpretive constructions derived from our qualitative analysis. The categories used throughout the Results are analytical labels developed to capture recurring patterns in participants’ observed and reported behaviors. As such, they function as conceptual tools that illuminate how participants appeared to enact and adapt evaluation practices within the distinct affordances of different technological environments. With this analytical framing in mind, we now describe the patterns observed across the two platforms.

Participants applied similar evaluation strategies when using both Google and Bing-Chat, but the implementation of these strategies differed between the platforms, with GenAI users demonstrating different tendencies than those described by the literature. When cognitive biases were at play, however, the differences between the platforms blurred: participants who relied on cognitive biases in Google did so also on Bing-Chat.

All thirty participants engaged in conversation with the chat, each asking at least two questions related to the topic under investigation, and many asking considerably more questions. All thirty participants were included in every stage of analysis; references to smaller numbers throughout this section reflect how many participants demonstrated or mentioned a particular behaviour or theme rather than any exclusion from the dataset. Below is an application of evaluation strategy themes when using both technologies.

### 4.1 Critical evaluation strategies applied in the two technologies

**Fig 2:**
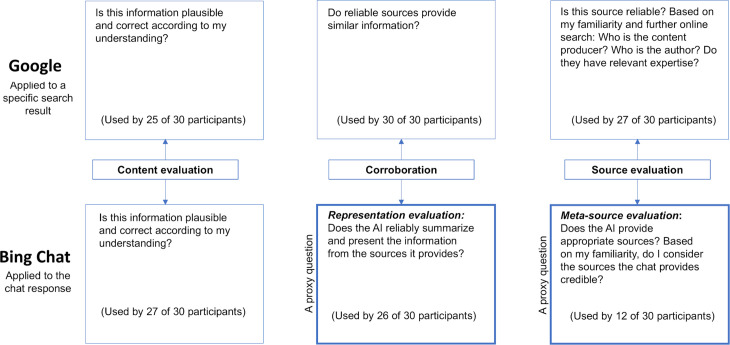
Critical evaluation strategies and their application using a search engine and GenAI.

In examining the critical evaluation strategies used by participants when interacting with Google and Bing-Chat, we focused on three main approaches: evaluation of the content itself (first-hand evaluation), corroboration, and evaluation of the source (second-hand evaluation). The findings show that while participants employed these evaluation strategies in both Google and Bing-Chat, their application of these methods varied considerably between the two technologies (Fig 2).

#### 4.1.1 Application of critical evaluation strategies when using Google.

When using Google, participants employed many of the critical evaluation strategies in ways that mirrored recommended practices found in the literature to examine specific websites appearing in search results. Given Google’s centrality as a global information retrieval tool, we use participants’ performance within this technology as a baseline.

Content evaluation strategy*.* A large majority of participants (25 of 30) relied on prior knowledge and assessed the plausibility of the information. This evaluation process, being primarily a mental exercise, surfaced during interviews when participants described their existing knowledge and reflected on how they used it. In addition, participants commented on their assessment of how ‘reasonable’ the information appeared and whether it was plausible. For instance, P1, a dancer with a high school level education, explained, ‘Based on my previous knowledge and intuition, I assess whether the content makes sense.’ P2, a nutrition science undergraduate, explained, ‘I based my evaluation on my knowledge from nutrition studies, I understand that a detox diet is unhealthy and, as a result, do not recommend it.’

Evaluation of information based on the degree of agreement with expert knowledge was not part of the search process of any participant. It was observed that none of the participants proactively sought information about the scientific consensus on their SSIs to evaluate the content they found. Two participants referred to this strategy in the interviews but did not actively use it during the task. P3, a data science undergraduate, understood from her search that ‘the scientific community has not yet conducted studies that confirm the issue one way or the other. So, it’s like I don’t know the exact answer either.’ While she acknowledged the importance of knowing the degree of agreement within the scientific community, she did not search for it independently via another information source.

Corroboration. During the observations, we saw that all participants consulted at least three different sources when searching with Google. We note that the prominent use of proactive corroboration might have been influenced by the research setting, possibly due to biases related to social interaction during the study [50]. Nevertheless, the consistent reference to this strategy in the interviews indicates participants’ awareness and understanding of its importance. All participants mentioned the strategy in their interviews and explained their preference for cross-referencing. P4, a physiotherapy undergraduate, explains, ‘I always go into more than one source, at least three sources, so that I can authenticate the information and cross-check it from different sources*.*’

Source evaluation*.* This was observed during Google searches in two ways: (1) evaluating the site or type of site, e.g., media source, or the Ministry of Health website; (2) evaluating the content’s author, e.g., author’s professional credentials. Evaluating the site type was particularly evident during observations, where 23 participants accessed official sources such as healthcare providers, the Ministry of Health, or academic articles, even when those were not the first search results. In the interviews, it was seen that site evaluation was based on familiarity with the specific site (27 participants), and awareness of commercial interests (26 participants). P4, a physiotherapy undergraduate, explained that she generally avoids sources she considers less reliable, stating*,* ‘…all kinds of videos on YouTube that people make where they seem to be trying to brand themselves, or ads, […] banners that pop up, Instagram, all kinds of things. I don’t view the information from these sources as something reliable.’ Indeed, P4 used only official sources, such as healthcare providers, during her performance task.

Evaluating the content’s author was discussed more in the interviews than observed. During the observations, only four participants specifically addressed the author’s identity and background. For example, P5, a computer science undergraduate, checked who authored the article he was reading and emphasized using the mouse that the author was a certified nutritionist. A proactive search for information about the author, as recommended in the literature, was observed only in the case of P6, a professional development coordinator in the high-tech sector. P6 also said in her interview: “I looked and saw that the person who wrote the article on the healthcare site wasn’t just anyone—she had professional expertise.” During the interviews, 26 participants mentioned the importance of the author’s expertise and experience in the relevant field. P7, a nutrition undergraduate, stated that she examines ‘whether the person who wrote the article is a clinical dietitian. If that is the case, then I would rather rely on it than if it were written by a naturopath.’

In sum, the use of critical evaluation strategies—content assessment, corroboration, and source verification—was prevalent and effectively integrated when searching on Google. Against this backdrop, we now describe critical evaluation strategies when engaging with information on Bing-Chat.

#### 4.1.2 Application of critical evaluation strategies when using Bing-Chat.

We found expressions of all the critical evaluation strategies when using Bing-Chat. Only content evaluation, however, was conducted similarly to Google, while corroboration and source evaluation strategies were implemented differently.

Content evaluation strategy. Most participants (27 of 30) assessed the GenAI’s responses by drawing on their prior knowledge or evaluating the plausibility of the information it provided. P6, a Hi-Tech professional development coordinator, explained her reasoning: ‘Logically speaking... it just feels unrealistic to me; I don’t feel that it is possible to escape from WIFI radiation.’ In Bing-Chat, as with Google, evaluating information based on the degree of agreement within the scientific community was not evident in the search processes. It was observed that none of the participants proactively asked the GenAI about the scientific consensus on the SSIs they were exploring. Two participants mentioned building on the degree of agreement between experts during their interviews, but they did not actively investigate it and relied on Bing-Chat’s content. For example, P8, a data science undergraduate, stated, ‘Bing-Chat repeatedly states that there is no scientific evidence supported by studies.’

Corroboration. Corroboration emerged as a crucial strategy in Bing-Chat. Having said that, the choice of the sources against which participants verified the data was not done independently by the users. The users adapted this strategy, asking themselves a proxy question: ‘Does the AI reliably summarize and represent the information in the sources it provides?’ rather than ‘Do reliable sources tell me the same thing?’ We termed this strategy ‘representation evaluation.’

This form of representation evaluation was common: it was observed that half of the participants actively clicked the sources provided by Bing-Chat to verify the AI-generated content against the information in those sources. The significance of this strategy also became evident during the interviews, especially when participants reflected on their ability to assess the content provided by Bing-Chat. P9, a computer science undergraduate, stated, ‘Bing-Chat provides the links, which is very convenient because I can go into them and check if they confirm the information that the chat provided’, as he did during the performance task. P10, a physiotherapy undergraduate, who clicked the links that the chat provided and read the additional content, added in the interview, ‘I relied on the sites that the chat directed me to, less on Bing-Chat’s answers.’ Only four participants did not refer to this strategy at all, either in observations or interviews.

Source evaluation strategy. On Google, users typically invested time in assessing a website’s credibility and its author’s expertise. However, on Bing-Chat users tended to use what we termed ‘meta-source evaluation’—judging Bing-Chat’s reliability and credibility based on the appropriateness of the sources it referenced, such as news outlets or academic sites. This method of source evaluation, as a form of reflective thinking, emerged during interviews where participants explained how they determined the reliability of the chat as an information source. P5, a computer science undergraduate, remarked, ‘It’s true that the chat gave me sources, but it gave me [names of news sites], which is not what I wanted. I was looking for scientific sources, not [media].’ P4, a physiotherapist undergraduate, echoed this sentiment, and elaborated, ‘I don’t trust Bing-Chat because it selects poor sources for me, so I can’t rely on it.’

Participants found it challenging to assess Bing-Chat’s reliability and credibility directly. Hence, some chose a proxy method—evaluating the sources it provided (Fig 2). Nevertheless, only 12 of the 30 participants mentioned this type of meta-source evaluation. None of the participants actively asked Bing-Chat about the sources’ expertise or the reasons for their inclusion. If they clicked on the sources, it was done to validate the content of the summary provided, not to evaluate the source itself.

### 4.2 Reliance on cognitive biases in the two technologies

All participants employed critical evaluation strategies, but some also relied on cognitive biases when assessing the information. Two biases were especially prominent: confirmation bias and the machine heuristic. Participants who exhibited confirmation bias in Google displayed the same tendency when using Bing-Chat. The machine heuristic, in contrast, was very visible only when using Bing-Chat.

#### 4.2.1 Confirmation bias.

Confirmation bias manifested similarly across both technologies among the same 16 participants when searching for information on the same topic. During the performance task, P13, a young adult with a high school education and currently unemployed, selected the disposable utensils SSI. The dilemma was: Is it environmentally better to use disposable, biodegradable utensils made of bamboo, or recyclable plastic disposable utensils? Although reusable utensils were not mentioned in the dilemma, before conducting any search, P13 initially expressed a strong belief that only reusable utensils should be used, and then proceeded to search for information relevant to the dilemma. After reviewing his final source from Google and forming a recommendation, he noted, *‘*But for sure, these [disposable bamboo utensils] are harming Earth. I have to find some disadvantages [for using them].’ He then refined his search by typing ‘bamboo utensils disadvantages.’ In the end, he recommended reusable utensils to his friend, consistent with his original belief. While searching on Bing-Chat, P13 encountered similar content supporting the environmental benefits of bamboo utensils. It was observed that to further confirm his beliefs, he deliberately guided Bing-Chat by asking, ‘What are the disadvantages of bamboo utensils?’

Similarly, in her performance task, P14, a medical secretary with a high school education, read about the use of Ritalin among children on an official healthcare website and stated: ‘I can’t believe that the American Association recommends Ritalin treatment for children as young as four. I’m almost shocked by this. The article doesn’t convince me at all. There’s nothing reassuring about it. There are no solid studies, and anyone can post things online... nothing has changed my opinion. I would suggest [my friend in the vignette] to explore natural alternatives instead.’ Despite finding expert sources during her online search, P14 ignored them because they conflicted with her pre-existing beliefs, which opposed the use of Ritalin. When searching for information on Bing-Chat, P14 formulated prompts that led to information emphasizing the negative aspects of using Ritalin: ‘What are the side effects of Ritalin?’ She ultimately decided, ‘I don’t recommend taking Ritalin due to its serious side effects. I would opt for alternative medicine and therapies that can address the issue. I believe in choosing natural solutions over artificial ones.’ These examples illustrate how confirmation bias drives the search and content evaluation similarly in both technologies.

#### 4.2.2 Machine heuristic.

A dozen participants implemented this heuristic when using Bing-Chat, assuming that automatic machines are more objective than people. This heuristic was expressed in a reflective assessment during interviews, where participants explained why they trusted the information from the chat. P13, an unemployed young adult with a high school education, pointed that ‘This technology knows everything. What do I know compared to it?’ When we asked P15, a social work undergraduate, if she doubted the information provided by the chat, she answered, ‘Not really, maybe because it’s a search engine specifically for these things. I don’t know, I just really trusted it.’ The machine heuristic was identified solely during evaluating information provided by Bing-Chat and was not observed when assessing information on Google. While participants trusted Google to provide quality sources in the high ranked results it provides, they did scroll down and review the other ones, deciding which to view. This bias might arise from the characteristics of the chat interface, as the chat itself generates the content. As stated by P11, an unemployed young adult with a high school education: ‘It never occurred to me to go into the sources it gave; I simply trusted it.’

In summary, the findings indicated that while participants employed established evaluation strategies across both platforms, their application was reshaped by the affordances of GenAI. Participants adapted familiar practices, such as corroboration and source evaluation, into new forms, including ‘representation evaluation’ and ‘meta-source evaluation’, reflecting efforts to interpret information within a system where the “source” was algorithmic and partly opaque. At the same time, reliance on cognitive shortcuts such as the machine heuristic revealed enduring epistemic limitations when interacting with AI-generated information. Together, these patterns demonstrated both continuity and transformation in users’ evaluative practices, highlighting how GenAI environments reconfigure traditional approaches to assessing information credibility.

## 5. Discussion

### 5.1 Adapting existing evaluation strategies for assessing AI-generated information

Evaluation of the content was similar across both platforms, but corroboration and source evaluation took on a different form when applied to Bing-Chat. The novelty of the evaluation strategies presented here lies in their distinctive manifestations within an AI-mediated environment. These have not been previously described in the literature and are revealed here due to the unique intersection of cognitive and sociotechnical inquiry.

Corroboration emerged as an essential strategy, with half of the participants using the sources provided by Bing Chat to verify that the AI-generated content matched the information in those sources. Users often relied on what we term ‘representation evaluation,’ asking themselves a proxy question: Does the AI accurately summarize and represent the information from the sources it provides? This is different from the original meaning of corroboration, which asks whether reliable sources confirm the same information independently. Representation evaluation embodies a form of epistemic delegation, in which users transfer the responsibility of verifying information to the AI, effectively trusting it to serve as a proxy evaluator of external sources.

The problem with this adaptation is that faulty answers may rely on summaries of unreliable sources, leading to unsupported claims and spreading misinformation. Echo chambers that limit exposure to conflicting beliefs [2] may curate their own corpus of conspiratorial sources. A similar risk could arise in GenAI, if it provides users with information based on such sources, since the selection of sources against which participants verified the data was not done independently. Users might perceive Bing-Chat providing specific sources as an implicit endorsement of them, even though the technology does not necessarily verify their reliability.

The most significant difference was observed in the application of source evaluation. When using Google, participants evaluated the expertise and integrity of specific websites and their authors; in Bing-Chat, however, participants engaged in what we term ‘meta-source evaluation.’ They evaluated the GenAI as a source based on the credibility of the sources it listed, but without evaluating these sources themselves. They assessed the sources only based on prior knowledge of the site’s name and domain. Meta-source evaluation represents processes of credibility transfer, in which the perceived trustworthiness of the AI interface is conferred onto the external sources it presents or cites. This finding aligns with Kresin et al. [59], who found that when evaluating scientific information on social media, participants cited familiarity with the source as a primary criterion for credibility. The familiarity criterion is so dominant that it can inhibit the use of additional credibility criteria.

These adaptations illustrate a partial shift from individual verification toward technologically mediated trust. Although participants described relying on evaluation strategies across both technological environments, these strategies did not transfer unchanged to the GenAI context. Instead, the participants’ evaluative practices were reconfigured to involve a growing dependence on algorithmic intermediaries for epistemic judgment.

Accordingly, established models of online information evaluation appear only partially applicable in AI-mediated environments. The move from a retrieval-based architecture (Google) to a generative, synthesized interface (Bing-Chat) reshaped not only how information was accessed but also how credibility was constructed. This limited transfer highlights the need to reconceptualize evaluation competencies in information science and to reconsider how such competencies are cultivated within science education.

### 5.2 Platform interface shapes user evaluation processes

Many differences in applying the critical evaluation strategies resulted from the two platforms’ user interfaces. The features of each triggered the use of different evaluation strategies.

Although users were often found to focus only on the first few links on Google and demonstrate high confidence in its ranking system, their interaction still required active epistemic agency, such as selecting sources, scanning multiple pages, and making judgments about relevance and credibility. After selecting a source, evaluating the information becomes more nuanced and differs across users. In contrast, Bing-Chat users first encounter the synthesized content, followed by a limited list of cited sources, a feature not shared by all GenAI systems (e.g., ChatGPT, which typically provides no citations at all). Consequently, unlike when using Google, most participants initially evaluated the information itself rather than its source. This inversion of the traditional evaluation sequence departs from theoretical perspectives such as Allchin’s [60] “who speaks for science” framework and Osborne and Pimentel’s [17] heuristics, both of which emphasize source-based evaluation as central to credibility assessment.

Bing-Chat’s conversational interface effectively centralizes epistemic authority within the system. This interface-driven behaviour exemplifies epistemic dependence on the AI, in which users delegate parts of their critical reasoning to automated intermediaries that appear both efficient and authoritative [29]. Consequently, Bing-Chat transformed not only users’ search behaviours but also their patterns of trust. While Google’s search architecture preserves a distributed, source-based model of evaluation, prompting users to navigate among multiple actors and texts, Bing-Chat’s generative architecture cultivated a mono-source illusion, positioning the AI itself as an epistemic gatekeeper. This shift illuminates how GenAI technologies potentially diminish users’ cognitive responsibility for evaluating information.

We should not ignore the growing blurring between the technologies, as Google increasingly presents knowledge graphs, displaying content directly on the search page using AI. Presenting the content at the top of the search results page may be pushing searchers into using a content evaluation strategy. Notwithstanding, however, only one participant in our study, P17, a computer science undergraduate, relied primarily on the information presented at the top of the Google results page and on the ‘people also ask’ features. All other participants chose sources and accessed them directly to obtain the information. This is changing as technology evolves. A 2025 Pew Research Center [61] study analyzed Google user behavior in the context of AI overviews appearing in search results. The data, collected from 900 American adults who agreed to share their browsing data, show that users are less likely to click on result links when an AI overview appears. In addition, users are less likely to click on sources cited within the overview itself and are more likely to end their browsing session after viewing a page containing an AI overview.

The contrast between Google and Bing-Chat can be better understood through the lens of interface affordances, which shape not only how users access information but also how they interpret and trust it [29] AI systems construct dynamic environments of affordances that both enable and constrain user cognition [29]. Bing-Chat’s conversational interface, which includes presenting synthesized responses before revealing their sources, embodies a design that prioritizes fluency and efficiency at the expense of transparency. This presentation structure fosters the machine heuristic, the tendency to over-rely on outputs perceived as competent or authoritative [14], thereby encouraging users to delegate elements of critical evaluation to the AI itself. In contrast, Google’s search architecture demands active navigation among multiple sources, maintaining a more distributed and deliberative model of epistemic engagement. Together, these dynamics highlight that evaluation strategies may be shaped less by user disposition than by platform design, as interface characteristics mediate the negotiation of epistemic trust and the distribution of cognitive responsibility in AI-mediated information environments.

Kresin et al. [59] argue for the development of unique educational frameworks for different technological platforms, specifically social media platforms. Our findings demonstrate that while participants were proficient in using specific information-evaluation strategies on one platform, this expertise did not necessarily transfer to another platform. This highlights the need to develop evaluation frameworks tailored to the GenAI information environment, while accounting for the epistemic limitations of this technology [62].

Importantly, the contribution of this study extends beyond interface effects alone. While affordances shape how users encounter and navigate information, our findings illuminate how individuals make epistemic inferences under conditions of algorithmic source opacity, where the origins, selection logic, and synthesis of information are partially obscured. The evaluation patterns observed, therefore reflect not only design features, but also users’ attempts to negotiate credibility and epistemic responsibility in environments where traditional source cues are displaced or reconfigured by generative systems. These epistemic conditions, rather than interface design alone, generate new demands for how evaluation skills should be conceptualized and taught.

### 5.3 The missing heuristic: An ongoing dialogue with GenAI about its answers and sources

GenAI has advanced the role of AI in dialogue-based applications [63]. Dialogue enhances the quality of AI-provided information and support by personalizing interactions based on dialogue history and adaptive responses [64]. Moreover, it can also help detect errors. In an experiment with preservice physics teachers, ChatGPT provided an incorrect answer to a challenging physics question, and participants were required to guide it through dialogue to recognize and correct its subtle errors in an otherwise well-constructed response [65].

Although all participants engaged in dialogue with the chat, each asking at least two questions related to the topic under investigation, and many asking considerably more, they did not engage in deeper verification practices. Unlike their behaviour on Google, where they often questioned the reliability of sources or compared information across multiple results, in the chat environment, they tended to accept the responses as given. Whereas dialogic interaction has the potential to serve as a metacognitive mechanism for testing claims and probing uncertainty, most participants treated the chat’s dialogue as functional rather than epistemic.

**Fig 3:**
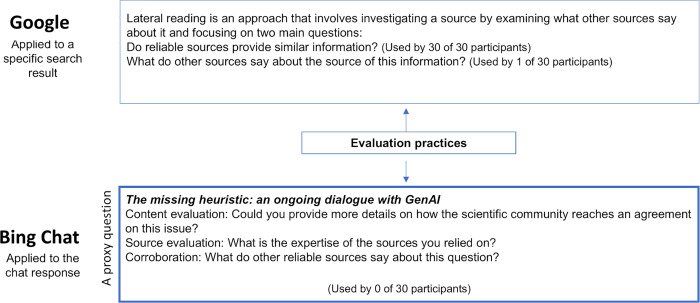
Evaluation practices and their application using a search engine and GenAI.

A key practice in information evaluation is lateral reading [21], which involves opening multiple tabs to verify a source’s credibility through independent web sources. Our findings suggest that this technique is less applicable and intuitive when working with GenAI—even if it provides its sources, one is not likely to apply lateral reading to assess them. When participants sought information within a chat interface, they often relied on the chat’s responses without deeply investigating the sources and also without interrogating the bot about its sources, their credibility, and how it chose them (Fig 3).

Engaging in dialogue with GenAI could enhance users’ ability to critically evaluate information. Assessing AI-generated information could leverage its dialogue-based nature. Ideally, users should be able to prompt the AI to provide more nuanced information, e.g., asking, ‘What evidence supports this claim?’ to evaluate the content. They could ask about the consensus in the field, e.g., ‘Does the scientific community agree on this issue?’. A dialogue-based heuristic could support source evaluation by asking, ‘What is the expertise of the sources you relied on?’, and corroboration, e.g., ‘What do other reliable sources say about this question?’. Dialogue-based evaluation techniques might equip users with essential skills to critically assess information within GenAI, aiding in the transition from traditional search engines to AI-based information exploration. We found that none of our participants used it, although it was presented to them during the familiarizing session before the main task (Fig 1).

### 5.4 Bias awareness: A missing element in science education

All participants used critical evaluation strategies, though some also relied on cognitive biases. Similar to other studies (e.g., [2,13,14]) on online information evaluation and human–computer interaction, the respondents in this study also relied on two main biases: confirmation bias, seen in both Google and Bing-Chat, and the machine heuristic, observed only in Bing-Chat. These biases can lead individuals to fail to fully gather, internalize, or systematically process information, even when they have the ability to do so [1].

Public understanding of GenAI is slowly emerging, as people begin to use these technologies [49]. The machine heuristic was common among our participants who received Bing-Chat provided information and assumed that the machine must be right. These echoes recent work showing that people with minimal experience using GenAI were also less aware of its epistemic limitations [66]. While knowledge of AI and its underlying mechanisms is critical to addressing the machine heuristic, awareness of this cognitive bias is key, as it empowers individuals to counteract it [67]. Confirmation bias, another common phenomenon in online environments [13], was found to be pervasive regardless of the technology used—whether participants accessed a web source or generative AI. As with the machine heuristic, awareness of this cognitive bias is essential, as it can help individuals to mitigate its effects [67].

Learning to recognize cognitive biases is crucial for scientific reasoning, yet it is often excluded from science curricula [68]. Many people are unaware of the cognitive factors shaping their perception of information. Educating individuals to recognize these influences, alongside content and source evaluation, is critical for their epistemic vigilance [67,69].

### 5.5 Limitations and way forward

Several limitations should be considered when evaluating the results. First, given the interpretative nature of qualitative coding, the findings inevitably reflect the researchers’ analytic lenses and theoretical commitments, even though systematic procedures were employed to enhance credibility. As such, other researchers might generate partially different categorizations from the same data. Furthermore, the performance task took place in a lab setting, likely increasing participant effort and introducing bias, as actions may align with perceived expectations [50]. This controlled environment lacks the complexity of real-world contexts, where information is usually evaluated in response to personal needs. However, a lab setting was chosen because it allowed for the collection of rich behavioural and verbal data under controlled conditions. Compared to surveys or online experiments, it enabled in-depth, real-time observation of participants’ engagement with information. At the same time, participants used their own personal devices, supporting a relatively authentic user experience compared to artificial setups involving standardized computers or restricted browsing interfaces. This balance between control and ecological validity offered a valuable compromise: while the setting may have heightened participants’ awareness of being observed, it ensured consistency across sessions and yielded nuanced insights into evaluative processes that are rarely observable in everyday contexts.

Additionally, a significant portion of the data is based on the participants’ self-reports regarding their information evaluation processes. This reliance on self-reporting is inevitable, as these processes involve internal thoughts that are not observable by an external observer. Furthermore, while generalizations are not warranted with small samples, such samples are appropriate for identifying and illustrating new phenomena, which is the primary contribution of our paper. We do not claim to generalize the frequency or prevalence of the strategies observed; rather, we highlight their existence.

Most participants had little prior experience with GenAI, so their strategies may reflect initial experimentation rather than stable evaluation practices. Moreover, the study was conducted in a single country and focused on a relatively narrow age group, which may limit the transferability of the findings across cultural or demographic contexts. In addition, given the nature of the study, we could not draw conclusions about the correlation between scientific and algorithmic knowledge and the ability to evaluate information across different platforms. Future research could adopt a quantitative approach to examine these relationships. A follow-up study could explore whether instructing participants in a dialogic approach to generative AI improves their ability to engage with it effectively.

## 6. Conclusion

As epistemic responsibility is progressively delegated to algorithmic systems, established models of credibility assessment and verification in scientific communication are being reshaped, underscoring the need to reconceptualize these notions in AI-mediated knowledge environments. Our findings demonstrate that evaluation practices developed in search-engine environments do not automatically transfer to AI-mediated contexts. Rather than simply applying familiar strategies in a new interface, participants adapted the existing evaluation strategies of corroboration and source evaluation to representation evaluation and meta-source evaluation when using GenAI. These novel adaptations reveal how practices of epistemic trust and verification are being reshaped in AI contexts, highlighting evolving processes of epistemic delegation and credibility transfer between users and generative systems. Together, they offer nuance to the current theoretical understanding of how people negotiate authority, accountability, and reliability when the information source itself is algorithmic.

The theoretical contribution has educational implications. As GenAI systems increasingly mediate access to scientific information, learners must critically assess not only what information is presented but also how it is generated. Educational efforts should therefore promote reflective evaluation practices, including examining the origins and credibility of AI-generated claims, assessing the cited sources, rather than assuming their credibility and relevance, and being aware of one’s automation bias.

Several concrete pedagogical and policy pathways can strengthen epistemic vigilance in AI-mediated environments. First, epistemic dialogue with GenAI could be explicitly integrated into science and digital-literacy curricula, fostering critical questioning as a habitual practice. These include teaching users to ask “What evidence supports this claim?” to evaluate content and “What is the expertise of the cited sources?” to evaluate sources. Second, designers and policymakers could collaborate to create AI interfaces that actively encourage users to engage in epistemic interrogation rather than consume information passively. This can be done, for example, by embedding explanatory information or by automatically suggesting additional source credibility information. By cultivating more discerning, dialogic, and epistemically responsible interactions with AI systems, societies can better navigate the opportunities and challenges of an era in which the boundaries between human and machine knowledge are continuously redrawn.

## Supporting information

S1 File Sample recruitment scheme aimed at diversifying academic level, science background, and algorithmic experience. (DOCX)

S2 File Sample characteristics. (DOCX)

S3 File Familiarity protocol: Presenting Bing-Chat to research participants prior to the performance task. (DOCX)

S4 File Ten vignettes to choose from in the second part of the performance task. (DOCX)

S5 File Observation protocol. (DOCX)

S6 File Interview protocol. (DOCX)
