# Correction: When the source is a bot: How people adapt their evaluation strategies to assess AI-generated content

**DOI:** 10.1371/journal.pone.0348712

**Published:** 2026-04-30

**Authors:** Shakked Dabran-Zivan, Inbal Klein-Avraham, Ayelet Baram-Tsabari

The references 7, 16, 49, 62, 66 and 69 were incorrectly written. Please see the correct references 7, 16, 49, 62, 66 and 69 below.

7. Klein-Avraham I, Dabran-Zivan S, Baram-Tsabari A. Do you know sometimes the information is wrong? Knowledge about and usage of generative artificial intelligence in the early adoption phase [presentation]. 26th Annual Conference of the ISCA; 2024; Herzliya, Israel.

16. Rozenblum Y, Dalyot K, Baram-Tsabari A. People who have more science education rely less on misinformation—even if they do not necessarily follow the health recommendations. J Res Sci Teach. 2025; 62(3):825−68

49. Greussing E, Guenther L, Baram-Tsabari A, Dabran-Zivan S, Jonas E, Klein-Avraham I, et al. Mapping cross-national patterns: The use of generative AI in science-related information retrieval across seven countries [accepted presentation]. Public Communication of Science and Technology (PCST) China Symposium; 2024 Oct; Suzhou, China.

62. Klein-Avraham I, Greussing E, Taddicken M, Dabran-Zivan S, Jonas E, Baram-Tsabari A. How to make sense of generative AI as a science communication researcher? A conceptual framework in the context of critical engagement with scientific information. JCOM. 2024;23(06):A05.

66. Klein-Avraham I, Baram-Tsabari A. Less epistemic knowledge about AI is associated with greater trust in AI and greater use of AI-generated information [conference presentation]. 75th Annual International Communication Association (ICA) Conference; 2025; Denver, CO, USA.

69. Feinstein NW, Baram-Tsabari A. Epistemic networks and the social nature of public engagement with science. J Res Sci Teach. 2024;61(9):2049–2068.
