# Supplementary material for: When the source is a bot: How people adapt their evaluation strategies to assess AI-generated content
Source: PLoS One. 2026 Mar 30;21(3):e0345300. doi: 10.1371/journal.pone.0345300 (PMC13035123; doi:10.1371/journal.pone.0345300)
Supplement: S1 File — (DOCX) [file pone.0345300.s001.docx]

# **S1. Sample recruitment scheme aimed at diversifying academic level, science background, and algorithmic experience.**

|  | Science background | |
| --- | --- | --- |
| Academic level | High | Low |
| Bachelor’s degree students with academic experience in algorithmics | Computer science or data science students with a science education academic level (n=5) | Computer science or data science students with NO science education academic level (n=5) |
| Bachelor’s degree students with NO academic experience in algorithmics | Science education, physiotherapy, and nutrition science students (n=5) | Education (excluding STEM), and social worker students (n=5) |
| High school graduates with NO academic experience in algorithmics or an academic level education | Professionals with a scientific background and relevance, e.g., medical secretary, Hi-Tech professional development coordinator (n=5) | Professionals with No scientific background, e.g., dancer, day-care staff (n=5) |
