# Supplementary material for: When the source is a bot: How people adapt their evaluation strategies to assess AI-generated content
Source: PLoS One. 2026 Mar 30;21(3):e0345300. doi: 10.1371/journal.pone.0345300 (PMC13035123; doi:10.1371/journal.pone.0345300)
Supplement: S2 File — (DOCX) [file pone.0345300.s002.docx]

# **S2. Sample characteristics****.**

| **Pseudonym** | **Gender** | **Recruitment group** (see note) | **Age group** | **Highest level of education** | **Professional or academic background** | **The dilemma chosen in the second part of the task** |
| --- | --- | --- | --- | --- | --- | --- |
| P1 | M | 6 | 23-29 | High school | Dancer | Hormonal treatment during menopause |
| P2 | M | 2 | 23-29 | Undergraduate student | Nutrition Science | Use of disposable utensils |
| P3 | F | 4 | 23-29 | Undergraduate student | Data Science | Radiation from Wi-Fi routers |
| P4 | F | 2 | 23-29 | Undergraduate student | Physiotherapy | Ritalin for children |
| P5 | M | 3 | 23-29 | Undergraduate student | Computer Science | Genetically Modified Food |
| P6 | F | 5 | 23-29 | High school | Hi-Tech professional development coordinator | Radiation from Wi-Fi routers |
| P7 | F | 2 | 23-29 | Undergraduate student | Nutrition Science | Child vaccinations |
| P8 | M | 4 | 23-29 | Undergraduate student | Data Science | Cellular radiation |
| P9 | F | 4 | 23-29 | Undergraduate student | Computer Science | Radiation from Wi-Fi routers |
| P10 | F | 2 | 23-29 | Undergraduate student | Physiotherapy | Use of disposable utensils |
| P11 | F | 6 | 18-22 | High school | Unemployed | Use of disposable utensils |
| P12 | F | 5 | 18-22 | High school | Waitress | Use of disposable utensils |
| P13 | M | 6 | 18-22 | High school | Unemployed | Use of disposable utensils |
| P14 | F | 5 | 30-39 | High school | Medical secretary | Ritalin for children |
| P15 | F | 1 | 23-29 | Undergraduate student | Social Work | Ritalin for children |
| P16 | M | 1 | 23-29 | Undergraduate student | Education | Aluminium in deodorant |
| P17 | M | 3 | 18-22 | Undergraduate student | Computer Science | Use of disposable utensils |
| P18 | F | 1 | 30-39 | Undergraduate student | Education | Ritalin for children |
| P19 | F | 1 | 23-29 | Undergraduate student | Education | Water fluoridation |
| P20 | M | 3 | 23-29 | Undergraduate student | Software Engineering | Radiation from Wi-Fi routers |
| P21 | F | 3 | 23-29 | Undergraduate student | Data Science | Ritalin for children |
| P22 | F | 1 | 30-39 | Undergraduate student | Education | Use of disposable utensils |
| P23 | M | 3 | 23-29 | Undergraduate student | Computer Science | Wind turbines |
| P24 | M | 4 | 23-29 | Undergraduate student | Computer Science | Genetically Modified Food |
| P25 | M | 4 | 23-29 | Undergraduate student | Data Science | Use of disposable utensils |
| P26 | F | 5 | 18-22 | High school | Artist | Radiation from Wi-Fi routers |
| P27 | F | 2 | 30-39 | Undergraduate student | Science education | Ritalin for children |
| P28 | F | 6 | 18-22 | High school | Unemployed | Use of disposable utensils |
| P29 | F | 6 | 18-22 | High school | Day care assistant | Ritalin for children |
| P30 | F | 5 | 18-22 | High school | Waitress | Aluminium in deodorant |

Note: 1. Education and social worker students; 2. Science education, physiotherapy and nutrition science students; 3. Computer science or data science students with an academic science education; 4. Computer science or data science students without an academic science education; 5. High school graduates with a matriculation certificate and scientific expertise but no academic education; 6. High school graduates with a matriculation certificate, no academic education, and lacking scientific expertise
