# Supplementary material for: When the source is a bot: How people adapt their evaluation strategies to assess AI-generated content
Source: PLoS One. 2026 Mar 30;21(3):e0345300. doi: 10.1371/journal.pone.0345300 (PMC13035123; doi:10.1371/journal.pone.0345300)
Supplement: S3 File — (DOCX) [file pone.0345300.s003.docx]

# **S3. Familiarity protocol: Presenting Bing-Chat to research participants prior to the performance task.**

The activity took place during the first Zoom meeting and lasted about 10 minutes.

**

We are going to introduce you to a recently released tool in Microsoft's search engine, Bing, called Bing-Chat.

**Have you heard of it or used it before?**

**If not:** Please open the Edge browser (from Microsoft) and share your screen. We'll be happy to introduce you to the tool.

**If so:** Great! Please open it on your device and share your screen so we can make sure we are on the same page before the performance task.

[The participant shares their screen using Edge]

**Next steps:**

1. Please click on the search window, but don't type anything yet.
2. Note the search suggestions that appear. At the top, you should see "Hello, I'm Bing —this is Copilot, your AI-based assistant on the Internet”. Now, click on "Start working."
3. Take a moment to explore the page. [The researcher remains silent for up to two minutes to allow the participant to explore the technology independently and observe different conversational styles.]

**Once the participant finishes exploring:**

Let's play around with the chat. In the window below, type any question that comes to mind, as you would in a conversation. You can ask anything you'd like. When you're ready, press Enter.

After the participant asks a question and receives a response, allowing them time to explore the interface further.

Excellent! Notice that the sources of the information are displayed here. You can ask additional questions. If you reach the quota limit (which you can see here), simply click on ‘New topic’ to start a new conversation.

**Do you have any questions?**

Now, let's try searching for information related to a scientific question from everyday life. For example, ask: "Which is better, a cup of coffee or a glass of wine?" Remember, you can ask follow-up questions in the chat. Go ahead and give it a try.

[allowing 2-3 minutes for the activity]

**Wrap-up:**

Okay, we'll meet again in [specify time—within an hour to a week later].

******

**In case of technical problems:**

1. Check the Edge browser settings—go to "About" to verify it's the latest version.
2. Use the chat bubble embedded within Edge itself.
3. If none worked, Bard by Google was used (in two cases)
