# Supplementary material for: When the source is a bot: How people adapt their evaluation strategies to assess AI-generated content
Source: PLoS One. 2026 Mar 30;21(3):e0345300. doi: 10.1371/journal.pone.0345300 (PMC13035123; doi:10.1371/journal.pone.0345300)
Supplement: S4 File — (DOCX) [file pone.0345300.s004.docx]

# **S4. Ten vignettes to choose from in the second part of the performance task.**

1. Genetically Modified Food:

A close friend who values her health but is facing financial difficulties approaches you with a dilemma: Is it better to avoid genetically modified foods to reduce potential health risks, or is it more important to maintain a diverse diet, even if it includes genetically modified ingredients?

What is the best advice you can give her?

1. Use of Disposable Utensils:

A close friend who cares about the environment but is facing financial difficulties approaches you with a dilemma: Is it better to use disposable, biodegradable utensils made of bamboo or plastic disposable utensils that can be recycled in the recycle bin?

What is the best advice you can give her?

1. Child Vaccinations:

A close friend, who has a 3-year-old daughter, has not vaccinated her child yet. Now, she wants to enroll her daughter in the best kindergarten in the area, a "vaccinated kindergarten" requiring a vaccination record. The friend turns to you with a dilemma: Are there childhood vaccines she should avoid, or should she complete all the vaccinations intended for 3-year-olds?

What is the best advice you can give her?

1. Water Fluoridation:

A close friend who is facing financial difficulties has heard that fluoride is added to the drinking water. She turns to you with a question: Is it better to consume more expensive mineral water to reduce potential health risks, or should she drink fluoridated water and benefit from its advantages?

What is the best advice you can give her?

1. Radiation from Wi-Fi Routers:

A close friend, a mother of two children aged 8 and 10, is debating which nearby school to send her children to. She turns to you with a dilemma: Should she send her children to a school without a Wi-Fi network to reduce potential health risks from exposure to radiation from wireless internet routers, or is it better to send them to the best school in the area, which does have a Wi-Fi network?

What is the best advice you can give her?

1. Wind Turbines:

A close friend, facing financial difficulties, is debating whether to move to a town near a wind turbine farm. The friend turns to you with a dilemma: Should she move and enjoy lower rent in a community that suits her, or should she look for another place to reduce the potential impact of the wind turbines on life quality?

What is the best advice you can give her?

1. Ritalin for Children:

A close friend has an 8-year-old daughter who has been diagnosed with Attention Deficit Disorder (ADD), which makes it difficult for her to keep up with schoolwork and integrate socially. The doctor has provided a prescription for Ritalin and left the decision to take the medication up to your friend. Your friend turns to you with a dilemma: Should she give her daughter Ritalin to help her function better at school and overall, or should she avoid medical treatment to reduce the potential harm from frequent and regular use of stimulant medications?

What is the best advice you can give her?

1. Cellular Radiation:

A close friend, facing financial difficulties and needing to move to a new apartment, shares that she found a spacious apartment in excellent condition, in a great location, and with relatively low rent. However, a cellular antenna is installed on the balcony above it. The friend turns to you with a dilemma: Should she rent this apartment, which meets all her needs and fits her limited budget, or should she take a smaller, more expensive apartment to reduce potential health risks?

What is the best advice you can give her?

1. Hormonal Treatment for Menopause:

Your beloved aunt, who has reached the age of 47, tells you she is suffering from bothersome hot flashes and frequent and extreme mood swings. A doctor she consulted explained that if she chooses, she can receive hormonal treatment to help her cope with the symptoms she described. Your aunt asks for your opinion on the matter: Should she receive hormonal treatment, or should she avoid it to reduce potential health risks?

What is the best advice you can give her?

1. Aluminum in Deodorant:

A close friend who values her health but is facing financial difficulties approaches you with a dilemma: She is debating whether to buy aluminum-free deodorant, which is more expensive than deodorant with aluminum. She wonders if aluminum-free deodorant is really more beneficial for her health.

What is the best advice you can give her?
