# Supplementary material for: When the source is a bot: How people adapt their evaluation strategies to assess AI-generated content
Source: PLoS One. 2026 Mar 30;21(3):e0345300. doi: 10.1371/journal.pone.0345300 (PMC13035123; doi:10.1371/journal.pone.0345300)
Supplement: S5 File — (DOCX) [file pone.0345300.s005.docx]

# **S5. Observation protocol.**

Guidelines for the researcher for a semi-participatory observation in the performance task.

**

The observer will **record in writing**: (1) what the participant is doing at any given time, including time stamps; (2) unclear or unreasoned actions, choices, and verbal remarks the participant made that require further explanations. These will be addressed at the beginning of the following interview.

Specifically, the observation will address and record the following aspects:

- To what degree does the participant cooperate with the different research tasks?
- How long did it take to look for information and reach a decision at each step?
- What does the searching process include in each of the four information-searching tasks, addressing:
  - How "invested" is the reading? (e.g., How much did people read from each source)
  - On Google: Did the queries included scientific jargon? How many results pages did participants look at? Did they open the different links in new tabs or windows? What links did they click on (paid, official sources, news, scientific, Wikipedia, commercial, social media, etc.)? How many alternative queries did they enter to refine their search? Did they look for information about the source/author for each link they opened?
  - On BingChat: How many prompts did participants use for each task? Did they develop a dialogue with the AI? Did the participant use scientific jargon? Did any of the prompts ask for clarification? Did the participant ask what the information was based on? Did the participant ask for additional sources?

At the end of each of the four information-search tasks:

- - Ask the participant: What did you decide?
    - Did the participant contemplate which decision to make and to what degree?
  - Regarding the outcome, ask the participants for their reasoning, i.e. Which considerations helped them form their decision?
    - Note (and clarify with the participant, if needed) to what degree their reasoning is based on scientific evidence or expertise of the sources.
    - Did the participant mention any sources/answers as guiding their response?

Recordings:

In addition to the observation, we record (1) the computer screen and (2) the audio throughout the experiment using the share screen option on the *Zoom* desktop app and an audio recorder.
