# Supplementary material for: When the source is a bot: How people adapt their evaluation strategies to assess AI-generated content
Source: PLoS One. 2026 Mar 30;21(3):e0345300. doi: 10.1371/journal.pone.0345300 (PMC13035123; doi:10.1371/journal.pone.0345300)
Supplement: S6 File — (DOCX) [file pone.0345300.s006.docx]

# **S6. Interview protocol.**

A semi-structured interview takes place after the performance task and a short break. The interview first addresses questions triggered by the observation, followed by questions regarding to the participant’s experience and perception of the information-searching process and outcomes. Not all the following questions were used in this analysis.

**

- Tell me, how was the experience? Evaluation of the overall experience.
- Did you learn something new? What was it? From what and about, what did you learn the most? How so?
- How satisfied are you with what you learned? And with your final decision?
- Thinking about the information you gathered, do you think you could use it to guide others in deciding on the issues (dilemmas) presented to you today?

*Regarding the first dilemma:*

- Are you satisfied with your final decision regarding the first dilemma? Are you satisfied with what you learned?
- Could you have used additional information (e.g., something you expected to find or a question you didn't get a satisfactory answer to)? If so, what is it?
- When you justified your decision, which sources did you mostly rely on?
- How accurate/correct do you think you are in your decision? What makes you think so?
- Was there information you needed but couldn’t find? Were there any other sources of information you would have asked?
- Did your background (what you learned in school, what you do at work, and your everyday experience) help you reach a decision? In understanding/evaluating the information?
- Please provide examples.
- Was there anything you wished you had learned to understand better the dilemma?

*Regarding the second dilemma:*

- Are you satisfied with your final decision regarding the first dilemma? Are you satisfied with what you learned?
- Could you have used additional information (e.g., something you expected to find or a question you didn't get a satisfactory answer to)? If so, what is it?
- When you justified your decision, which sources did you mostly rely on?
- How accurate/correct do you think you are in your decision? What makes you think so?
- Was there information you needed but couldn’t find? Were there any other sources of information you would have asked?
- Did your background (what you learned in school, what you do at work, and your everyday experience) help you reach a decision? In understanding/evaluating the information?
- Please provide examples.
- Was there anything you wished you had learned to understand better the dilemma?

*For both:*

- How did you decide whether you should trust the results on Google? Which indicators did you use?
- (If the participant is lost, we can add: how did you choose which site to click on?)
- What made you doubt a search result after you clicked it? Which indicators did you use? (How did you know to do that? Where did you learn to do that?)
- How did you decide whether you should trust the answers BingChat provided?
- What indicated reliability or trustworthiness? (or bias?)
- What made you doubt the information BingChat provided?
- Does the decision you made on both dilemmas coincide with the perceptions, values, and ideology that you had when you first came here today? (If not), what made you change your mind? (if yes) what could have changed your mind?

*Let’s compare the two technologies:*

- Which information tool, Google or BingChat, was more convenient for you to use? Why?
- With which technology was it easier to evaluate the reliability of the information? Why?
- Using which technology was the process more (1) sufficient (2) effective (3) productive? Please explain why and provide examples
- In your opinion, what are the limitations of each of the technologies that you used today when searching for information about science?
- Which of these tools is more practical for everyday use? Which one of the technologies will you use yourself, if any?
- Which technology makes you feel empowered and capable of making the decision?
- Which technology will you recommend to others? To which users might it be more helpful? For what purposes?
- If you were asked to do this exercise all over again, using only one of these technologies, which would you prefer? Why?

*[if not covered in previous questions, add also]: Let’s talk a little specifically on BingChat*

- Does using BingChat make it feel like you can search for information easily or quickly?
- Does the information you got from BingChat seem trustworthy? Does it seem accurate? What led you to think so?
- Generally, do you think one can trust the information provided by BingChat? why so?
- Is BingChat convenient for independent learning? What makes you say that?
- Does using BingChat make you feel capable of making an informed decision? In what way?
- Can you say what you like better in BingChat, compared to Google? What makes it so?
